# Smoking Cessation Apps for Smartphones: Content Analysis With the Self-Determination Theory

**DOI:** 10.2196/jmir.3061

**Published:** 2014-02-12

**Authors:** Jounghwa Choi, Ghee-Young Noh, Dong-Jin Park

**Affiliations:** ^1^Hallym UniversityChuncheonKorea, Republic Of

**Keywords:** medical informatics applications, smoking cessation, communication media

## Abstract

**Background:**

Smartphones are increasingly receiving attention from public health scholars and practitioners as a means to assist individuals’ health management. A number of smartphone apps for smoking cessation are also available; however, little effort has been made to evaluate the content and functions of these apps employing a theoretical framework.

**Objective:**

The present study aims to analyze and evaluate the contents of smoking cessation apps available in South Korea employing the self-determination theory (SDT) as a theoretical framework for analysis. This study analyzes the extent to which smoking cessation apps have features that satisfy the basic needs identified in the SDT, which stimulate autonomous motivation. The type of motivational goal content manifested in the apps and how the goal content was framed are also explored. By assessing the features of smoking cessation apps based on the SDT, this study aims to offer direction for improvement for these apps.

**Methods:**

Out of 309 apps identified from the iTunes store and Google Play (excluding 27 duplications), 175 apps were randomly drawn and analyzed. The coding scheme was drafted by the authors based on the SDT and gain/loss framing theory and was further finely tuned through the process of coder training and by establishing intercoder reliability. Once the intercoder reliability was established, the coders divided up the rest of the sample and coded them independently.

**Results:**

The analysis revealed that most apps (94.3%, 165/175) had at least one feature that tapped at least 1 of the 3 basic needs. Only 18 of 175 apps (10.3%) addressed all 3 basic needs. For goal content, money (53.7%, 94/175) showed the highest frequency, followed by health (32.0%, 56/175), time (7.4%, 13/175), and appearance (1.1%, 2/175), suggesting that extrinsic goals are more dominantly presented in smoking cessation apps. For the framing of goal content, gain framing appeared more frequently (41.7%, 73/175).

**Conclusions:**

The results suggest that these smoking cessation apps may not sufficiently stimulate autonomous motivation; a small number of apps addressed all 3 basic needs suggested by the SDT (ie, autonomy, competence, and relatedness). The apps also tended to present extrinsic goal content (primarily in terms of money) over intrinsic ones (ie, health) by primarily adopting gain framing. Implications of these findings for public health practitioners and consumers are discussed.

## Introduction

### Background

New information technologies are being used to assist continuous health behavior change and the management of chronic disease [1,2]. Smartphones are receiving increasing attention as a potential information technology. Because of the portability of smartphones, which enables people access to them 24 hours a day, long-term management and reinforcement of health behaviors through a variety of communications and apps becomes possible. Based on the recognition of this potential, a variety of smartphone apps for smoking cessation have been released. The problem, however, is that apps developed by individuals are being distributed extensively and it is hard to see if these apps were actually developed based on theoretical and scientific evidence. It is possible that apps with unverified contents have an adverse effect on the health of citizens. For example, a news report argues that many smartphone apps for depression could actually produce countereffects by fostering depression rather than offering a cure for it [3]. Only a few academic studies address this issue [4-6], and little effort has been made in evaluating the contents and functions of the apps employing theoretical framework.

To address this issue further, the present study aimed to analyze and evaluate the contents of smoking cessation apps. We used the self-determination theory (SDT) [7,8] as a theoretical framework for analysis. Although popular health behavior theories, such as the health belief model [9] or the theory of reasoned action [10], are limited in addressing the mechanism underlying the maintenance of changed behaviors [11], SDT offers an insight into this issue, which is critical to smoking cessation [12,13]. According to SDT, the types of motivation that drive individuals’ behaviors can vary on the degree of autonomy; if behaviors are triggered by autonomous motivation rather than controlled motivation, people are more likely to engage voluntarily in behavioral changes, which in turn are more likely to be sustained. A number of empirical studies support the effect of autonomous motivation on long-term behavior change, which lasts for a year or longer [14-17]. Therefore, smoking cessation apps with features and contents that are configured by SDT are expected to be more effective.

We analyzed the extent to which smoking cessation apps have features that satisfy the basic needs identified in SDT, which in turn stimulate autonomous motivation. The types of motivational goal contents manifested in the apps and how the goal contents are framed were also explored. By assessing the features of smoking cessation apps based on SDT, this study offers direction for improvement for these apps.

### Self-Determination Theory

#### Overview

According to SDT, gratification of 3 basic psychological needs—autonomy, competence, and relatedness—is essential to the development of intrinsic or autonomous motivation and the maintenance of behavioral change [7,18]. The following discussion will examine how these basic psychological needs could be applied to health behaviors, especially to smoking cessation behavior.

#### Autonomy

The need for autonomy refers to the individual’s need for regulating their behaviors based on their own values, interests, or satisfaction for a behavior itself, not by external influences [18]. SDT research has proposed that social environmental factors, such as autonomy support, play an important role in inducing autonomous motivation by satisfying one’s need for autonomy. The effects of autonomy support are evidenced in smoking-related research. According to studies by Williams et al [15,19], an autonomy-supportive communication style induced more autonomous motivation for smoking cessation than a non-autonomy-supportive one. This effect is not only for the short term, but also is likely to last for long-term smoking cessation.

Autonomy support is characterized by the following: (1) providing meaningful reasons for a behavior, (2) providing choices and alternatives for a behavior, (3) supporting individuals’ initiatives, and (4) acknowledging individuals’ perspectives (eg, negative affect regarding difficult behaviors) [20,21]. To this regard, we can expect that smoking cessation apps with such characteristics would bear greater effect on smoking cessation.

#### Competence

The need for competence refers to the individual’s need for feeling competent about themselves and improving their skills or talents [18]. SDT explains that experience of self-confidence and capabilities related to a behavioral change is necessary for autonomous motivation. The positive effect of competence has been found in studies on smoking cessation [21,22]. These studies showed that competence, along with autonomy, increases the probability of maintaining long-term smoking cessation. These findings are in-line with previous studies, which have reported that self-efficacy or perceived control plays an important role in smoking cessation [23,24].

A sense of competence would be formed and supported by skills, tools, or pertinent feedback that assist individuals to implement behavioral changes as well as to overcome obstacles that inhibit behavioral changes [25]. Therefore, smartphone apps would promote competence for smoking cessation with features such as: (1) providing informational resources concerning skills and knowledge for smoking cessation, (2) providing supporting tools for behavioral implementation, and (3) providing feedback on behaviors implemented and on the individual’s progress.

#### Relatedness

The need for relatedness concerns feelings connected to others [18]. According to SDT, relatedness is important for autonomous motivation because it promotes the internalization of extrinsic causes; individuals may voluntarily engage in behavioral change because of someone else who means a lot to them, even though the behavior by itself is not interesting. In fact, the importance of relatedness, noted as “social support,” has been evidenced in various domains of health behaviors, such as breast cancer [26], human immunodeficiency virus (HIV) [27], and exercise [28]. The positive effect of social support on smoking cessation has also been reported in several studies [29-31].

Social support is now available in cyberspace owing to advancements in information technology. For example, various forms of online communities, such as chat rooms, weblogs, or bulletin board systems, can provide social support related to health behaviors [26,32]. The up-and-coming social media is also expected to contribute to social capital formation by enabling individuals to connect with one another [33]. Smartphone apps are often developed in conjunction with social media or offer functions to access communities online. By taking advantage of these characteristics, smoking cessation apps are expected to satisfy relatedness.

In summary, autonomy, competence, and relatedness are important factors to induce intrinsic motivation for long-term behavioral changes. Smoking cessation apps addressing these basic needs are expected to have greater effects on changing smoking behavior. To this regard, this study proposes the first research question: To what extent do smoking cessation apps have features that contribute to the satisfaction of the basic needs (ie, autonomy, relatedness, and competence)?

### Goal Contents and Smoking Cessation Behavior

According to SDT, the way individuals select and internalize goals for their own life affects the individual’s behavior and psychological well-being. SDT distinguishes intrinsic goal contents (eg, personal growth, health, affiliation) from extrinsic goal contents (eg, financial success, fame, physical attractiveness) [34]. Previous research suggested that intrinsic goals, compared to extrinsic goals, have a more positive and long-term impact on behavioral changes by activating autonomous motivation [35-37]. The advantage of intrinsic goal contents remains significant irrespective of individuals perceiving an activity as instrumental for intrinsic or extrinsic goal attainment [38]; even when individuals pursue extrinsic goals for autonomous reasons, the negative effect of extrinsic goal aspiration remains significant [18]. Regarding smoking cessation, studies have indicated that personalized feedback works better than financial incentive to increase the cessation rates and to prevent relapse [39]; the higher the extent to which one craves physical health as a goal, the greater the readiness to quit smoking or the higher the rate of maintaining a nonsmoking status compared to pursuing cessation for extrinsic reasons (eg, immediate reinforcement such as saving money) [40-42] and those who receive monetary compensation are less likely to remain nonsmokers for long compared to those without monetary compensation [43]. For this reason, Ryan and Deci [8] expressed concern about the use of controlling mechanisms, such as monetary benefits, to promote behavioral changes.

Given the relationship between goal content and long-term behavioral change, it is important to examine what type of goal content is suggested in smoking cessation apps. In smoking cessation apps, the goal content can be implied by presenting the consequences of behaviors. For example, many smoking cessation apps provide a function that calculates monetary gain or loss from smoking or not smoking cigarettes, whereas some provide a function that calculates health consequences, such as life time lost from smoking or blood concentration of carbon monoxide. The former can be viewed as presenting extrinsic goals in terms of the financial aspect, whereas the latter present intrinsic goals in terms of health. Therefore, this study proposes the second research question: To what extent is each type of goal content (ie, intrinsic vs extrinsic) presented in smoking cessation apps?

### The Gain/Loss Framing of Goal Contents and Smoking Cessation Behavior

Goal content can be presented differently depending on the type of framing employed. Accordingly, the impact of goal content may rely not only on the kind of goal, but also on the kind of framing employed to present the goal. Gain framing emphasizes the positive outcomes, either physical or psychological benefits, which will be gained from adopting the recommendations. Loss framing obtains compliance by emphasizing the negative consequences resulting from not adopting the recommendations. Pelletier and Sharp [44] argued that both intrinsic and extrinsic goals could be presented in a gain or loss frame. For example, a message may suggest that one can gain health by engaging in a behavior (ie, gain framing of an intrinsic goal) or that one can lose money by engaging in a behavior (ie, loss framing of an extrinsic goal).

It has been proposed that gain framing works better for prevention behaviors (eg, smoking cessation) and loss framing works better for detection behaviors (eg, mammography) [45]. Although some scholars argue for the meager effect of a gain-framed message for prevention behavior [46], gain framing’s advantage over loss framing for prevention behaviors is relatively robust [47-48]. Recently accumulated evidence, including Gallagher and Updegraff’s meta-analysis [49], provide support for this argument, at least for smoking cessation [50-54]. Given the evidence for the effectiveness of smoking cessation apps adopting gain-framed information, the third research question is put forth to assess the extent to which the apps adopt gain framing over loss framing in presenting the goal content: To what extent are gain and loss framing used in presenting the goal content implied in smoking cessation apps?

## Methods

### Sample

In this study, smoking cessation apps are defined as apps that aim to induce behavioral changes to quit smoking. Although app markets are not strictly bounded by region, an app available in one country may not be available in other countries. Therefore, we limited our analysis to the apps available in South Korea, which the authors had access to. To draw a sample, apps in Google Play and the Apple iTunes store, the largest open markets for smartphone apps, were searched during the last week of November 2013 utilizing the keywords “smoking” and “smoking cessation,” either in Korean or in English. Although the search from the iTunes store produced a definite number of cases for each keyword, the total number was not identifiable for Google Play (ie, expressed as +100,000). Therefore, for Google Play, the initial search result was sorted by relevance and then the Web pages were reviewed until a Web page without a smoking cessation app was found. A list of apps was compiled by examining the title and the description of apps searched. That is, for both the iTunes store and Google Play, we used an additive strategy (ie, adding apps to the list while reviewing the resulting search pages for each keyword), rather than a deductive strategy (ie, deducting the irrelevant apps from the definite number apps from the initial search) to compile the list of apps.

Among the apps searched with keywords, those which were not relevant to smoking cessation were excluded. Apps were also excluded even if they had some relevance to smoking cessation in the following cases: (1) task management apps were dropped unless their primary purpose was to aid smoking cessation; (2) hypnosis apps for smoking cessation were disregarded because they attempt to exert a subconscious influence and are not appropriate to be analyzed within the frame of SDT; (3) apps developed for physicians to aid their medical treatment, rather than for general consumers, were also not included; (4) apps offering simulation of smoking were also not included unless they clearly stated their purpose as smoking cessation. As a result, 309 apps were included: 167 from the iTunes store and 169 from Google Play, with 27 duplications identified. To draw a sample, 175 apps were randomly drawn based on sample size guidelines [55,56]. The apps searched and downloaded from December 2012 through January 2013 were analyzed.

### Coding of Apps

#### Coding Scheme

The draft of the coding scheme was developed based on the aforementioned conceptualization of each variable, driven by SDT and the gain/loss framing theory. Based on this draft, the authors reviewed a part of the smoking cessation apps and revised the draft, taking into account their characteristics. This version of the draft was further fine-tuned through the process of coder training and establishing intercoder reliability.

#### Descriptive Characteristics

To identify the general characteristics of the smoking cessation apps, the following items were coded: market type (Google Play, iTunes store, or available in both), price type (free vs paid), developer type (individuals or individual developer groups, nonprofit organizations, or companies), and contents type (information-centric, function-centric, or information-function balanced). The number of downloads was also included in the coding scheme; however, this data was collected for Android apps only because the iTunes store does not offer this information.

#### Autonomy

To assess autonomy, we examined whether an app had features related to the aforementioned autonomy-supportive characteristics. First, apps were examined to determine if they offered relevant information addressing reasons for stopping smoking (eg, scientific evidence on the risk of smoking). Apps were coded as "yes" on this if they offered a separate section for the information, otherwise they were coded as "no." That is, apps presenting a few sentences simply stating, “smoking is bad for health,” were coded as "no" on this. Second, apps were coded as "yes" if they offered functions allowing one’s own smoking cessation plan (eg, setting up a cessation schedule or amount of cigarettes to smoke; choosing cessation methods) to support the individual’s initiatives, the second aspect of autonomy support. These functions were also deemed as tapping the third aspect of autonomy support (ie, providing individuals choices and alternatives for smoking cessation behavior) because they allow individuals with different approaches to quit smoking using their own time frame and strategies. The last aspect of autonomy is acknowledging individuals’ perspectives or negative affects experienced from quitting smoking. This only can be assessed in the context of interpersonal communication; therefore, apps were not coded on this.

#### Competence

Competence is formed and supported by skills, tools, or pertinent feedback that assist individuals to implement behavioral changes [25]. Therefore, features related to competence were assessed in light of the absence (coded as "no") or presence (coded as "yes") of the following features in an app. First, apps were coded as "yes" if they offered information resources concerning skills and knowledge for smoking cessation; this included offering specific guidelines or tips for course of actions to quit smoking and resources to increase knowledge regarding smoking cessation (eg, FAQ, hotline, links to website, quiz or game to improve knowledge related to smoking cessation). Second, the absence or presence of tools assisting the implementation of behavioral change was assessed, which included functions to send alert or alarm messages to warn or remind, and functions to record and track one’s own quitting or smoking attempts. Finally, apps were coded as "yes" if they offered feedback functions, such as offering an analysis of one’s own quitting attempts or efforts or providing cues to progress or achievement toward smoking cessation.

#### Relatedness

Because relatedness concerns feelings connected to others, the apps were examined in terms of whether they offered functions that allowed for interacting with others or receiving social support from others. Specifically, the presence (coded as "yes") or absence (coded as "no") of the following attributes were coded: providing functions to interact with others (eg, online communities, social media), and providing functions to deliver social support messages (eg, recording and playing messages from family members or friends).

#### Goal Contents and Framing

To analyze the goal content and the gain/loss framing implied in the smoking cessation apps, we examined the consequences of quitting smoking that manifested in the functions of smoking cessation apps based on the initial identification by the authors. Specifically, the absence (coded as "no") or presence (coded as "yes") of each of the 4 types of goal content (eg, money, appearance, health, and time) were coded because apps often presented multiple goal content simultaneously. Each type of goal content identified was further examined to see if it was presented in terms of gain or loss. For example, when a function was identified as presenting money as a goal, it was examined to determine if it concerned calculating monetary loss from smoking or gain from quitting smoking. It was possible that an app could offer both gain-framed and loss-framed functions; thus, the presence (coded as "yes") or absence (coded as "no") of each function was coded. Gain/loss framing of other goal content type was also measured in the same manner: appearance (ie, a function that shows appearance deterioration from smoking or appearance improvement from quitting smoking), health (ie, a function that shows health-related loss from smoking or health-related gain from quitting smoking, such as life expectancy or blood pressure), and time (ie, a function that calculates time wasted from smoking/saved from quitting smoking). Money and appearance are deemed extrinsic goals and health is considered an intrinsic goal [18]. In all, 7.5% (13/175) of apps suggested time wasted or saved as a consequence of smoking or quitting smoking. However, no theoretical rationale was found for the category of time; thus, we did not categorize it into either category.

## Results

### Intercoder Reliability

Intercoder reliability was established following the guidelines by Lombard et al [57]. Two undergraduate students fluent in both English and Korean served as coders. They became acquainted with the coding scheme through 2 training sessions. Examining the apps based on the coding scheme, the coders were asked to try every component in an app. For example, when apps required users to enter their own information, create an account, or take a test, the coders did so to fully appreciate the features of the apps. It took the coders approximately 15 minutes to code a simple app and 30-40 minutes for a more complex app. For initial intercoder reliability, the coders independently coded 30 apps that were not part of the sample. Intercoder reliability was calculated with PRAM ver 0.4.5 [58]. For some items, the reliability was less than .70; thus, some of the initial coding categories were adjusted after discussion between the researchers and the coders through another training session and the coding scheme was finalized as described previously. For final intercoder reliability, 50 apps (28.6% of the total sample) were drawn from the sample. The percent agreement was greater than .90 for all coding items. The Cohen’s kappa or Scott’s pi coefficients were >.70 for most coding items. However, the coefficients were zero for loss framing of time and 2 attributes of relatedness (ie, connected to online communities, connected to social media). The coefficient for loss framing of appearance turned out to be negative. This could happen for binary variables in which 1 of the values (1 is present or 0 is absent) is observed very rarely or not at all because these coefficients take into account the prevalence of the categories [59]. An examination of the data revealed that no case in the sample data for intercoder reliability was coded on loss framing of time and 2 attributes of relatedness. As for the loss framing of appearance, both coders agreed the cases were absent of this feature, except for 1 case. In the process of resolving the difference, it was found that 1 coder missed the feature while reviewing the app, as opposed to judging the feature differently from the other coder. Because the percent agreement for those items exceeded .90 and it was possible for more cases to be observed in the whole sample, we decided to retain these items. The disagreement between the coders was resolved through a discussion with both coders and researchers. After establishing intercoder reliability, the coders divided up the rest of the sample and coded them independently.

### Descriptive Statistics of the Sample

The descriptive characteristics of the apps were examined in terms of market, price, developer, and content type (Table 1). Among 175 apps, 14 (8.0%) were found both in Google Play and the iTunes store. The rest were solely from either the iTunes store (45.7%, 80/175) or Google Play (46.3%, 81/175). Overall, free apps (55.4%, 97/175) were more frequently observed than paid apps (44.6%, 78/175). Most apps were developed by individual developers or developer groups (68.0%, 119/175). Only 9 apps (5.1%) were found to be developed by nonprofit organizations. For content type, most apps tended to be function oriented (60.0%, 105/175). Approximately one-fifth (18.9%, 33/175) of the apps were judged to balance function with information.

**Table 1:** Descriptive statistics of smoking cessation apps in the sample (N=175).

Analysis categories		Distribution, n (%)
**Market type**		
	Android	81 (46.3)
	Apple	80 (45.7)
	Android/Apple	14 (8.0)
**Price type**		
	Free	97 (55.4)
	Paid	78 (44.6)
**Developer type**		
	Individual or developer group	119 (68.0)
	Nonprofit organization	9 (5.1)
	Company	47 (26.9)
**Contents type**		
	Information oriented	37 (21.1)
	Function oriented	105 (60.0)
	Information and function balanced	33 (18.9)

### Features of Smoking Cessation Apps Related to Basic Needs

The first research question asked the extent to which smoking cessation apps have features related to the basic needs (ie, autonomy, relatedness, and competence). Our analysis of the coded data of the apps revealed that most apps (94.3%, 165/175) had at least one feature which tapped at least 1 of the 3 basic needs, whereas few apps (10.3%, 18/175) addressed all 3 basic needs. Calculation of the total basic need score by summing the scores on all 3 basic needs produced a mean of 2.82 (SD 1.56), indicating that smoking cessation apps have approximately 3 kinds of features tapping any of the basic needs.

At least one feature related to competence was found in most apps (86.9%, 152/175). These features included offering how-to information regarding smoking cessation (40.0%, 70/175), functions to record and track one’s own quitting or smoking attempts (34.3%, 60/175), or cues to achievement or progress (44.0%, 77/175). In regards to autonomy, approximately half (48.6%, 85/175) of the apps contained at least one feature supporting autonomy. The observed features tapping autonomy were information regarding reason for smoking cessation (33.7%, 59/175) and functions allowing one’s own smoking cessation plan (20.0%, 35/175). In contrast to competence and autonomy, a smaller proportion of the apps addressed relatedness (21.1%, 37/175). The most frequently observed feature related to relatedness was connection to social media (17.7%, 31/175), through which the users could share their own efforts to quit smoking and receive supportive messages from their acquaintances (Table 2).

**Table 2:** Analysis of smoking cessation app features related to basic needs (N=175).

Basic needs and app features			n (%)
**Autonomy**			
	Information addressing reason for smoking cessation		59 (33.7)
	Function allowing one’s own smoking cessation plan		35 (20.0)
	Offers at least 1 of the autonomy features above		85 (48.6)
**Competence**			
	How-to information and resources		70 (40.0)
	**Tools assisting implementation of behavior**		
		Functions to send alert or alarm messages to warn or remind	18 (10.3)
		Function to record and track one’s own quitting or smoking attempts	60 (34.3)
	**Offers feedback**		
		Analysis of performance for quitting attempts or efforts	50 (28.6)
		Cues to achievement or progress	77 (44.0)
	Offers at least 1 of the competence features above		152 (86.9)
**Relatedness**			
	**Function to interact with others**		
		Connected to online communities	7 (2.4)
		Connected to social media	31 (17.7)
	Function to offer or record messages to cheer up		2 (1.1)
	Offers at least 1 of the relatedness features above		37 (21.1)

### Goal Content and Framing in Smoking Cessation Apps

The second research question attempted to address the extent to which each type of goal content (ie, intrinsic vs extrinsic) was implied in the smoking cessation apps. Among the 4 types of goal content identified in the coding scheme, money (53.7%, 94/175) showed the highest frequency, followed by health (32.0%, 56/175), time (7.4%, 13/175), and appearance (1.1%, 2/175) (Table 3). When grouping money and appearance as extrinsic goals and health as an intrinsic goal, 29.1% (51/175) of the apps were found to present both extrinsic and intrinsic goals simultaneously. The apps suggesting only extrinsic goals were 25.7% (45/175), whereas the apps suggesting only intrinsic goals were minimal (2.9%, 5/175) (Table 4). This result suggests that extrinsic goals are more dominantly presented among smoking cessation apps than intrinsic goals.

The third research question concerned the framing of goal content implied in the smoking cessation apps. Analysis of the data revealed a more frequent appearance of gain framing. That is, the apps tended to focus on monetary gain (ie, amount of money saved from not smoking a cigarette) or health benefits (eg, life time earned or blood pressure decreased from quitting smoking) rather than monetary loss (ie, amount of money wasted from smoking a cigarette) or health loss (eg, life time lost or blood pressure increased from smoking) (Table 3).

**Table 3:** Implied goal content and framing type in smoking cessation apps available in South Korea (N=175).

Goal content and framing		n (%)
**Money**		94 (53.7)
	Gain only	57 (32.6)
	Loss only	25 (14.3)
	Gain and loss both	12 (6.9)
**Health**		56 (32.0)
	Gain only	38 (21.7)
	Loss only	14 (8.0)
	Gain and loss both	4 (2.3)
**Time**		13 (7.4)
	Gain only	8 (4.6)
	Loss only	2 (1.1)
	Gain and loss both	3 (1.7)
**Appearance**		2 (1.1)
	Gain only	0 (0.0)
	Loss only	2 (1.1)
	Gain and loss both	0 (0.0)

**Table 4:** Goal content (intrinsic vs extrinsic) of smoking cessation apps available in South Korea (N=175).

Category	n (%)
Intrinsic goal content (ie, health) only	5 (2.9)
Extrinsic goal content (ie, money and appearance) only	45 (25.7)
Intrinsic and extrinsic goal content both	51 (29.1)
Total number of cases suggesting goal content	101 (57.7)

**Figure 1:**
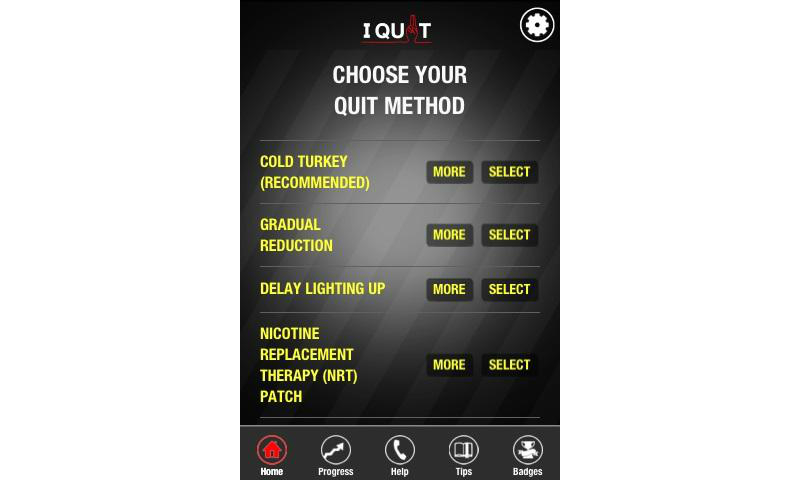
Screenshot of the HPB I Quit app by the Singapore Health Promotion Board.

**Figure 2:**
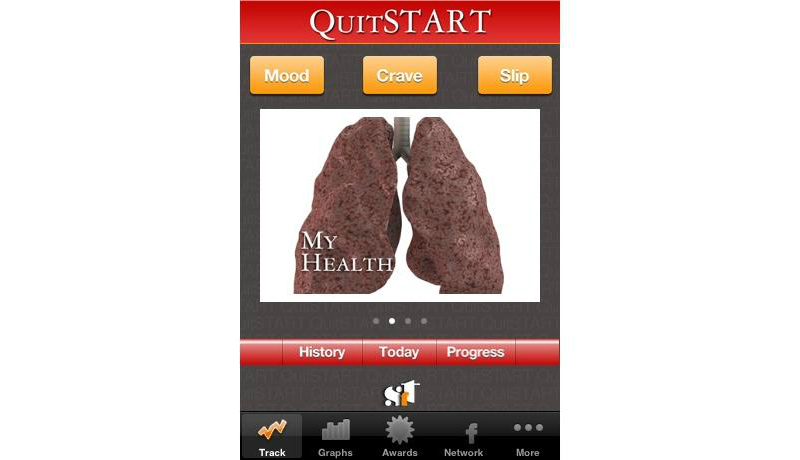
Screenshot of the QuitSTART app by the US Tobacco Control Research Branch at the National Cancer Institute.

**Figure 3:**
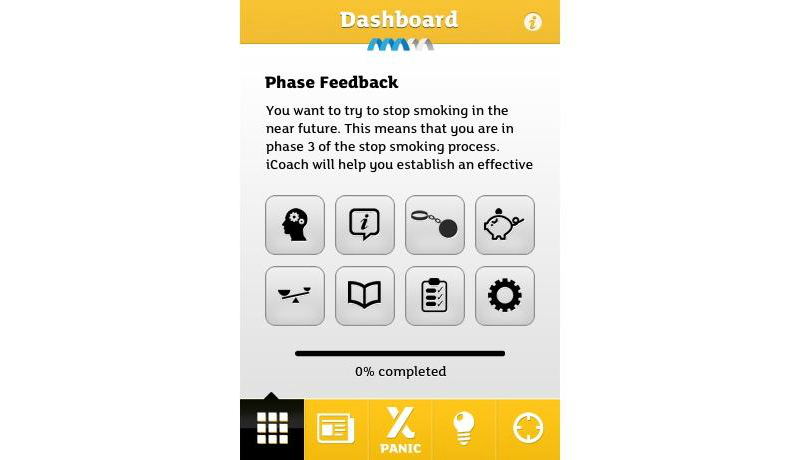
Screenshot of the FCB/Exsmokers iCoach app by the European Commission.

**Figure 4:**
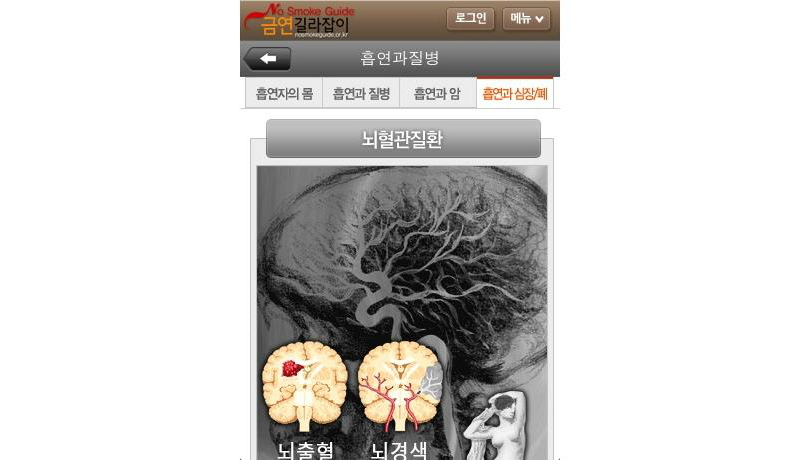
Screenshot of the [No Smoke Guide] app by the Korean Ministry of Health and Welfare.

Based on this analysis, we identified the top smoking cessation apps. First, we identified the apps that had at least one feature that tapped each of the 3 basic needs. In all, 18 apps met this criterion. These were then sorted by total SDT score, the sum of scores for each SDT attribute. Among the top-ranked apps, 4 apps developed in the public sectors are presented in Table 5 along with their unique features: Singapore’s Health Promotion Board (Figure 1), Tobacco Control Research Branch at the National Cancer Institute in the United States (Figure 2), the European Commission (Figure 3), and the Korean Ministry of Health and Welfare (Figure 4). They tended to contain common features, such as aiding one’s own quitting plan and management of it, providing tips and strategies, and allowing sharing through social media.

**Table 5:** Top-ranked smoking cessation apps developed in the public sector.

Name of app	Rank^a^	Market type	Unique features
HPB I Quit [60]	1	Apple/Android	Developed by the Singapore Health Promotion Board. Links to QuitLine and quit centers. Introduces various methods to quit smoking.
QuitSTART [61]	3	Apple	Developed by Tobacco Control Research Branch at the US National Cancer Institute. Offers symbolic signs of achievement; tracking, and analysis of behaviors.
FCB/Exsmokers iCoach [62]	3	Apple/Android	Developed by the European Commission. Aid quitting plan and managing it step-by-step; offers analysis of behaviors.
[No Smoke Guide]^b^ [63]	7	Apple/Android	Offered by the Korean Ministry of Health and Welfare. Offers information on quit centers; in-depth information on smoking and health.

^a^Rank score from top 10 list of apps that included paid and free apps.

^b^App in Korean (name of app translated into English).

## Discussion

### Principal Results

Health promotion through personal media, such as smartphones, is receiving increasing attention as a variety of health-related smartphone apps are being introduced in the market. However, only a few studies have evaluated the content and function of these apps by employing a theoretical framework. Therefore, the present study analyzed a representative sample of smoking cessation apps (N=175) accessible in South Korea based on the theoretical framework of SDT.

The analysis suggests that most apps contain at least 1 kind of feature related to at least 1 of the components of the basic needs proposed by SDT, primarily competence. A relatively small proportion of the apps, however, addressed all 3 basic needs (18/175, 10.3%). In particular, only a few apps supporting relatedness were found (37/175, 21.1%). SDT studies have shown that satisfaction of the 3 basic psychological needs is fundamental to meaningful and continuous behavioral change [7], and this is also supported by research in smoking cessation [15,21]. It might be reasonable to say that smoking cessation apps that lack 1 or more components of the basic needs may be relatively limited in producing positive effects on continuous behavioral changes compared to those satisfying all aspects of basic needs. The efficacy of the apps would depend on how individuals make use of them. Notwithstanding, it is expected that there is at least more potential that the apps with features addressing the 3 basic needs could be more efficacious.

The analysis of goal content implied in the functions of smoking cessation apps also reveals interesting findings. The most frequently observed type of goal content was money (an extrinsic goal) followed by health (an intrinsic goal). More than half of the smoking cessation apps (53.7%, 94/175) offered a function that calculated money earned or lost per cigarette. In addition, the smoking cessation apps tended to include either extrinsic goal content only or extrinsic and intrinsic goal content together. Given that extrinsic goal content is more likely to be related to controlled motivation, and controlled motivation is more likely to be induced even when both extrinsic and intrinsic goal content are suggested at the same time [64], these smoking cessation apps may be limited in activating autonomous motivation. Because behavioral changes prompted by controlled motivation are less likely to last, the efficacy of these smoking cessation apps in inducing long-term behavioral change is open to question.

For the analysis of framing of goal content, it was found that gain framing was more frequently adopted in suggesting goal content; apps tended to offer a function that calculated the financial or health gain from quitting smoking rather than money or health loss from smoking. As mentioned previously, gain framing has been found to be more effective than loss framing for prevention behaviors, such as quitting smoking [49]. Further, gain framing is more effective than loss framing in moving smokers toward the preparation stage from the contemplation stage of smoking cessation [65]. Those who download the smoking cessation apps might possibly be interested in quitting smoking or have decided to quit smoking (ie, those who are in the contemplation or preparation stage). In this sense, the fact that smoking cessation apps are adopting gain framing more than loss framing could be viewed positively.

We expect that the findings of this present study can be generalized to the United States and possibly to other countries as well. Out of the 309 apps that were the population of this study, 21 apps (6.8%) were available only in Korean, not in English. For the remaining apps that were available in English, when we searched the US Google Play and iTunes store with an anonymous helper with a US account, only a few apps (7/309, 2.3%) were not found. It seems that most of these apps are circulated globally, whereas a small portion of the apps may be available only to certain regions. If this is the case, the findings of this study might be sustained in other countries.

### Implications and Limitations

The present study is significant in that it conducted a theory-driven analysis using SDT to evaluate smoking cessation apps. SDT explains how individuals internalize external forces, make autonomous decisions, and implement voluntary actions. The SDT explanation of the 3 basic needs and their roles in the motivation process lends a useful theoretical framework to analyze and evaluate smoking cessation apps because the theory informs the kind of characteristics that a smartphone app aiming for long-term behavioral change needs to have to stimulate autonomous motivation. Although this study is not a direct test on the effects of smoking cessation apps, this theory-driven analysis allows an indirect assessment of smoking cessation apps available in the market.

In addition, the present study provides several practical implications for consumers, public health experts, and practitioners. In view of SDT, our study suggests that many of the smoking cessation apps available in the market may be partially limited in inducing long-term smoking cessation. It might be useful for consumers if public health practitioners or organizations evaluate the apps and recommend high-quality smoking cessation apps. Further, providing consumers with a guide regarding how to evaluate smoking cessation apps would be helpful. In addition, public health organizations could develop apps using the evaluation criteria addressed in this study.

The findings of the present study are bounded by several limitations. First, we recognize that our analysis is limited in fully analyzing the whole aspects of an app. For example, a function that is coupled with social media could enhance not only relatedness, but also competence because it is also possible that individuals exchange tips and information for smoking cessation through social media. Also, we primarily focused on the functional features of the apps and did not examine the messages delivered in the apps. Second, some apps had functional features that were not specified on the menu, but activated when used over the longer term. Because our coders did not use the apps for the long term, these features might not have been captured. Third, our study is limited in providing descriptions of all kinds of smoking cessation apps because we excluded hypnosis apps in our analysis. Future studies that examine hypnosis apps in terms of the messages delivered would provide a complementary picture about smoking cessation apps to this study. Finally, the inferences from the findings of this study could be limited because this study is not a direct test of the effects of smoking cessation apps. Rather, it draws an indirect inference regarding their efficacy based on previous SDT studies. It should be noted that the conclusion on the effect of gain or loss framing and the effect of goal content are still open to discussion. Although a substantial number of studies support the advantage of gain-framed messages over loss-framed messages in regards to smoking cessation, researchers also acknowledge that the effect of gain or loss framing could be complex, so that multiple mediators and moderators can play into the framing effect [47-49]. Future studies should address this issue by exploring the effects of smoking cessation apps through experimental studies.

### Conclusions

To sum up, the present study shows that smoking cessation apps have features that satisfy the basic needs to some extent; thus, they could be a useful tool to promote smoking cessation. However, it is also true that many of these apps provide limited features to satisfy all 3 basic needs and present extrinsic goals rather than intrinsic goals. As a result, they may serve as a limited tool in stimulating autonomous motivation for long-term smoking cessation. This finding is in-line with studies by Abroms et al [4,5] that concluded that smoking cessation apps, even popular ones, are far below the US Public Health Service’s Clinical Practice Guidelines for Treating Tobacco Use and Dependence. Although the present study is only a snapshot of smoking cessation apps presently available, this study suggests that there is still room to increase the efficacy of these apps and that a good theory, such as SDT, can guide the process. Public health practitioners would need to play a role in planning mobile health apps by informing developers of the theory.

## Abbreviations

SDT: self-determination theory
